# An Efficient, Parallelized Algorithm for Optimal Conditional Entropy-Based Feature Selection

**DOI:** 10.3390/e22040492

**Published:** 2020-04-24

**Authors:** Gustavo Estrela, Marco Dimas Gubitoso, Carlos Eduardo Ferreira, Junior Barrera, Marcelo S. Reis

**Affiliations:** 1 Center of Toxins, Immune-Response and Cell Signaling (CeTICS), Laboratório de Ciclo Celular, Instituto Butantan, Butantã, São Paulo-SP 05503-900, Brazil; estrela.gustavo.matos@gmail.com; 2 Instituto de Matemática e Estatística, Universidade de São Paulo, São Paulo-SP 05503-900, Brazil; gubi@ime.usp.br (M.D.G.); cef@ime.usp.br (C.E.F.); jb@ime.usp.br (J.B.)

**Keywords:** machine learning, supervised learning, information theory, mean conditional entropy, feature selection, classifier design, Support-Vector Machine, U-curve problem, Boolean lattice

## Abstract

In Machine Learning, feature selection is an important step in classifier design. It consists of finding a subset of features that is optimum for a given cost function. One possibility to solve feature selection is to organize all possible feature subsets into a Boolean lattice and to exploit the fact that the costs of chains in that lattice describe U-shaped curves. Minimization of such cost function is known as the U-curve problem. Recently, a study proposed U-Curve Search (UCS), an optimal algorithm for that problem, which was successfully used for feature selection. However, despite of the algorithm optimality, the UCS required time in computational assays was exponential on the number of features. Here, we report that such scalability issue arises due to the fact that the U-curve problem is NP-hard. In the sequence, we introduce the Parallel U-Curve Search (PUCS), a new algorithm for the U-curve problem. In PUCS, we present a novel way to partition the search space into smaller Boolean lattices, thus rendering the algorithm highly parallelizable. We also provide computational assays with both synthetic data and Machine Learning datasets, where the PUCS performance was assessed against UCS and other golden standard algorithms in feature selection.

## 1. Introduction

“With four parameters I can fit an elephant, and with five I can make him wiggle his trunk” is a well-known saying attributed by Enrico Fermi to John Von Neumann [1]. Indeed, that quote is a good allegory to the core aim of feature selection, which is to select elements from a set *S* of features for classifier design during supervised learning, in a trade off between the consideration of as many features as possible and the avoidance of classification error due to the lack of samples. As in the quote, to achieve such trade off, it is necessary to select features that are very informative about the relationship between observations and labels (as the four parameters that “fit an elephant”) at the same time discarding the ones that bring fewer information (as the parameter that makes him “wiggle his trunk”). To evaluate whether a subset X⊆S has only very informative features, a possibility is the usage of an information theory-based criteria such as the ones based on conditional entropy.

The conditional Shannon entropy (or simply conditional entropy) H(Y|X=x) is a measure of the probability mass concentration of a random variable Y given the occurrence of the random variable X realization x. If we average the values of *H* for all possible realizations of X, then we have the application of a mean conditional entropy (MCE) criterion. During a feature selection procedure, X and Y are random variables associated with, respectively, the subset X⊆S and the set of labels L={0,1,…,K} in the supervised learning problem. Hence, if |X| is the cardinality of *X*, then we can say that X is |X|-variate, with the domain of each of its features context-dependent (e.g., discrete or real-valued), whereas Y is univariate and draws from *L*. Therefore, during the evaluation of a subset *X* using a MCE criterion, one must estimate $\hat{E}\left[H\left(Y|X\right)\right]$ from data. However, the number of available samples might not be sufficient for good estimates. One way to circumvent the lack of samples is to penalize an underrepresented pair 〈x,y〉 that is jointly draw from X and Y (e.g., a pair that was observed only once); that approach was successfully tested in the designing of morphological operators using MCE as criterion [2]. Another possibility is the application of hypotheses about the relationship among features. For instance, the Correlation-Based Feature Selection (CFS) is a cost function which works under the assumption that good features are highly correlated with the class, yet uncorrelated with each other [3]. Thus, CFS is capable to deal with moderate levels of dependence among features [3]. There are other methods that rely on similar hypotheses, such as the minimum redundancy, maximum relevancy criterion [4]. However, those criteria fail to handle features with greater levels of interactions among them; two examples of the latter are the parity problem and the inference of intrinsically multivariate predictive genes [5,6].

Regardless of the considered criterion, a feature selection algorithm is required to evaluate the collection P(S) of all subsets of *S*, using a cost function $c:P\left(S\right)\mapsto R_{\geq 0}$ whose mapping is a feature selection criterion. Therefore, the goal is to find a subset X⊆S that minimizes *c*. This latter problem, also known as the *feature selection problem*, is NP-hard [7], which means that it belongs to the class of problems for which the existence of polynomial-time algorithms to solve them is unknown. Once exhaustive search for an optimum *X* may be unfeasible even for moderate sizes of *S* [8], there is (in the literature) a number of proposed feature selection algorithms, both optimal and suboptimal ones [8,9]. Among the many families of those algorithms, there are the sequential selection, genetic algorithms, and search algorithms that organize the search space as a graph. Sequential selection algorithms are suboptimal, greedy approaches where, given a subset X⊆S and for each s∈S\X, it evaluates c(X∪{s}) and updates *X* with the element *s* that minimizes c(X∪{s}). Although sequential selection approaches were proposed as early as in the 60’s [10], the most popular algorithm of that type, the Sequential Forward Floating Selection (SFFS), was introduced in 1994 by Pudil and colleagues [11]. Opposite to the Sequential Forward Search (SFS) [12], SFFS allows removal of elements, thus avoiding to get stuck in local minima [11]. Another important family of algorithms for feature selection is composed of evolutionary methods, among them the Genetic Algorithms (GAs) [13]. A very efficient GA algorithm is the “Cross generational elitist selection, Heterogeneous recombination, and Cataclysmic mutation GA” (CHCGA), whose complicated name describes its main properties [14]. Finally, a feature selection procedure that organizes the search space as a graph consider as a vertex each subset X⊆S and as an edge a pair of subsets (X,Y) such that X⊆Y⊆S and |X|=|Y|+1. Therefore, one can apply one of the many graph-search algorithms that are available in the literature; a widely used one for feature selection is the Best-First Search (BFS) [15].

The aforementioned graph representation of a search space in a feature selection procedure is isomorphic to a Boolean lattice L=(P(S),⊆), where any maximal chain is in the form $\emptyset \subseteq \left\{s_{1}\right\}\subseteq \left\{s_{1},s_{2}\right\}\subseteq \ldots \subseteq S$, where $s_{i}\in S$. Moreover, due to the phenomenon known as “curse of dimensionality” (i.e., the increase of selected subset *X* size without a corresponding increase in the number of samples), the associated costs of any chain in L, that is $c\left(\emptyset \right),c\left(\left\{s_{1}\right\}\right),c\left(\left\{s_{1},s_{2}\right\}\right),\ldots ,c\left(S\right)$, describe an U-shaped curve [16]. That important observation led to the development of a feature selection procedure based on the minimization of a cost function whose chains in L describe an U-shaped curve; such minimization problem is also known as *the U-curve problem* [16]. Following the introduction of the U-curve problem approach for feature selection, novel algorithms were proposed to tackle that problem; for instance, the improved U-curve Branch and Bound (iUBB) is a branch-and-bound algorithm for solving the U-curve problem that is very robust to noise; however, it has an explicit representation of the whole search space, which constrains the feasibility of this algorithm to small sizes of *S* [17]. More recently, a study proposed the U-Curve Search (UCS) algorithm, which is an improvement on the very first algorithm introduced to tackle the U-curve problem: while the latter was a suboptimal algorithm, UCS always returns an optimal solution [18]. UCS manages the search space as a Boolean lattice and controls the pruned elements of P(S) through collections of intervals in the form of either [∅,X]:={A∈P(S):A⊆X} or [X,S]:={A∈P(S):X⊆A}, where X⊆S. Search space exploration is done through a Depth-First Search (DFS) procedure that tests necessary and sufficient conditions to prune intervals without the risk of losing elements of minimum cost. However, although UCS is indeed an optimal algorithm for the U-curve problem, computational experiments with that algorithm showed that the required computational time to achieve an optimal solution was exponential on the number of considered features [18], which raised doubts about the nature of that issue: it could be due to algorithm design, to the intrinsic problem structure, or both.

In this work, we demonstrate that the scalability issue verified in the UCS algorithm actually arises from the fact that the U-curve problem is NP-hard. To mitigate this problem, we introduce a new optimal feature selection algorithm, the Parallel U-Curve Search (PUCS). PUCS includes a parallelization scheme that allows an improvement on the required computational time that is proportional to the available resources (CPU cores or threads). Results from computational experiments, either with artificial data or in MCE-based feature selection procedures, confirmed that PUCS can speedup the search for optimal solutions of the U-curve problem. The remainder of this article is organized as follows: in the Results section (Section 2), we formally introduce the U-curve problem and prove that it is NP-hard (Section 2.1), present the PUCS algorithm (Section 2.2) and also results from computational experiments with that new algorithm (Section 2.3). In the Discussion section (Section 3), we analyze the results from Section 2 and discuss possible directions in this research line. Finally, in Materials and Methods (Section 4), we detail the computational environment and the software used to carry out the experiments shown throughout this paper.

## 2. Results

In this section, we present the main contributions of this paper. We start by presenting a formal description of the U-curve problem, followed by a proof that it is actually NP-hard (Section 2.1). Since the solvability of NP-hard problems in polynomial time is an open problem, such proof means that the U-curve problem can be solved optimally only for small instances (what is “small” in this context is discussed in Section 2.3). In the sequence, we introduce the Parallel U-Curve Search (PUCS) algorithm, including its theoretical principles, a pseudocode and also an exemplary simulation on a Boolean lattice of degree five (Section 2.2). Finally, we provide results from computational assays, in which PUCS performance is assessed against other algorithms; to this end, we executed experiments on synthetic data and also in the feature selection step of classifier design (Section 2.3).

### 2.1. The U-Curve Problem and Its Computational Complexity

Let *S* be a non-empty set, and P(S) be the collection of all subsets of *S*, also known as the power set of *S*. A *chain* is a collection $\left\{X_{1},\ldots ,X_{|S|+1}\right\}$ of |S|+1 pairwise distinct elements in P(S) such that $X_{1}\subseteq \ldots \subseteq X_{|S|+1}$. A *cost functionc* is a function that takes values from the power set of *S* to the non-negative real numbers, that is, $c:P\left(S\right)\mapsto R_{\geq 0}$. Let *X* be an element in P(S). *X* is of *minimum cost* (or simply *minimum*) if there is no element *Y* in P(S) such that Y≠X and c(Y)<c(X). Those concepts allow us to define a decomposable in U-shaped curves cost function.

**Definition** **1.** *Let c be a cost function and S be a non-empty set. c is* decomposable in U-shaped curves *if for any chain X⊆P(S), it holds that if $X_{1}\subseteq X_{2}\subseteq X_{3}$, $X_{i}\in X$, then $c\left(X_{2}\right)\leq max\left\{c\left(X_{1}\right),c\left(X_{3}\right)\right\}$.*

A decomposable in U-shaped curves cost function is an extension of the class of set operators that are decomposable as the intersection of a decreasing and an increasing operators [19]. We can now state the problem that is addressed in this work.

**Problem** **1.** *(U-curve) Let S be a non-empty set and c a decomposable in U-shaped curves cost function. Find a subset X⊆S such that X is minimum.*

As it was stated in the previous section, Problem 1 can be used as an approximation for the more general feature selection problem. However, as we will demonstrate in the following, it is unlikely that that Problem 1 can be solved efficiently (i.e., in a polynomial time).

**Theorem** **1.** *The U-curve problem is NP-hard.*

**Proof.**  We start defining a decision problem version of the U-curve problem by choosing an integer *k* and asking whether there is a subset X⊆S such that c(X)≤k. We will show that this decision problem is at least as hard as the subset sum problem, a well-known NP-hard problem [20] (pp. 1013). In the subset sum problem, one has a finite set *W* of non-negative integers and a non-negative integer *t*, and wants to know if there is a subset Y⊆W such that the sum of the elements in *Y* is exactly *t*. Given an instance 〈W,t〉 of the subset sum problem, we will construct an instance 〈S,c,k〉 of the U-curve decision problem, in a time bounded by a polynomial in |W|. This instance is such that there is a subset Y⊆W whose sum is equal to *t* if and only if there is a subset X⊆S whose cost is less or equal to *k*. The set *S* is defined as a copy of *W*, the value of *k* is defined as zero, and the cost function *c* is:

(1) $$c\left(X\right)=\left|t\;-\sum_{x\in X}x\right|,\mathrm{for}\mathrm{all}X\subseteq S,$$

where |.| is the absolute value function. Now we will prove that *c* is indeed a cost function decomposable in U-shaped curves, that is, for each chain X⊆P(S), for any $X_{1},X_{2},X_{3}\in X$, $X_{1}\subseteq X_{2}\subseteq X_{3}$ implies that $c\left(X_{2}\right)\leq max\left\{c\left(X_{1}\right),c\left(X_{3}\right)\right\}$. Let us consider two cases. In the first case, let $X_{2}$ be an element of P(S) such that $\sum_{x_{2}\in X_{2}}x_{2}<t$. Thus, for any $X_{1}\subseteq X_{2}$ we have:

(2) $$c\left(X_{2}\right)=\left|t\;-\sum_{x_{2}\in X_{2}}x_{2}\right|\leq \left|t-\left(\sum_{x_{2}\in X_{2}}x_{2}\;-\sum_{x\in X_{2}\backslash X_{1}}x\right)\right|=\left|t\;-\sum_{x_{1}\in X_{1}}x_{1}\right|=c\left(X_{1}\right).$$

The second case, where $X_{2}$ is an element of P(S) such that $\sum_{x_{2}\in X_{2}}x_{2}\geq t$, is symmetrical to the first one. Thus, once either $X_{1}\subseteq X_{2}$ implies that $c\left(X_{2}\right)\leq c\left(X_{1}\right)$ or $X_{2}\subseteq X_{3}$ implies that $c\left(X_{2}\right)\leq c\left(X_{3}\right)$, it holds that $X_{1}\subseteq X_{2}\subseteq X_{3}$ implies that $c\left(X_{2}\right)\leq max\left\{c\left(X_{1}\right),c\left(X_{3}\right)\right\}$. Therefore, we conclude that the cost function *c* is decomposable in U-shaped curves.

Finally, we need to show that there is a subset Y⊆W whose sum is equal to *t* if and only if there is a subset X⊆S whose cost is less or equal to *k*:

⇒: If there is a subset Y⊆W whose sum is equal to *t*, then there is a subset X⊆S such that X=Y and, by the definition of *c*, the value of c(X) is 0≤k.

⇐: If there is a subset X⊆S such that c(X)≤k=0, then there is a subset Y⊆W such that Y=X and, by the definition of *c*, c(X)=0 implies that the sum of elements in *Y* is equal to *t*. □

### 2.2. The Parallel U-Curve Search (PUCS) Algorithm

We demonstrated that the scalability problem of the UCS algorithm is due to the fact that the U-curve problem (Problem 1) is NP-hard (Theorem 1). To circumvent that issue, we developed the Parallel U-Curve Search (PUCS) algorithm. PUCS solves the U-curve problem through a partitioning of the search space, which enables each part to be solved in an independent, parallelized way. Once each part is also a Boolean lattice, the algorithm can be called recursively on such smaller lattices. As the recursion base case, one might use the UCS algorithm itself or any other procedure, either an optimal or a suboptimal one. In the following, we will present some theoretical principles of the PUCS algorithm, as well as an example of the algorithm dynamics.

#### 2.2.1. Theoretical Principles of the PUCS Algorithm

For the sake of simplicity, from now on, we will denote a subset *X* of *S* through a binary string, where digit positions correspond to element positions in a lexicographical sorting, and the digit values 0 or 1 represent, respectively, absence or presence of a given element. For example, consider a set S={c,b,e,d,a}: the lexicographical order of *S* is 〈a,b,c,d,e〉, and the subset {b,e} can be represented with the string 01001.

**Figure 1:**
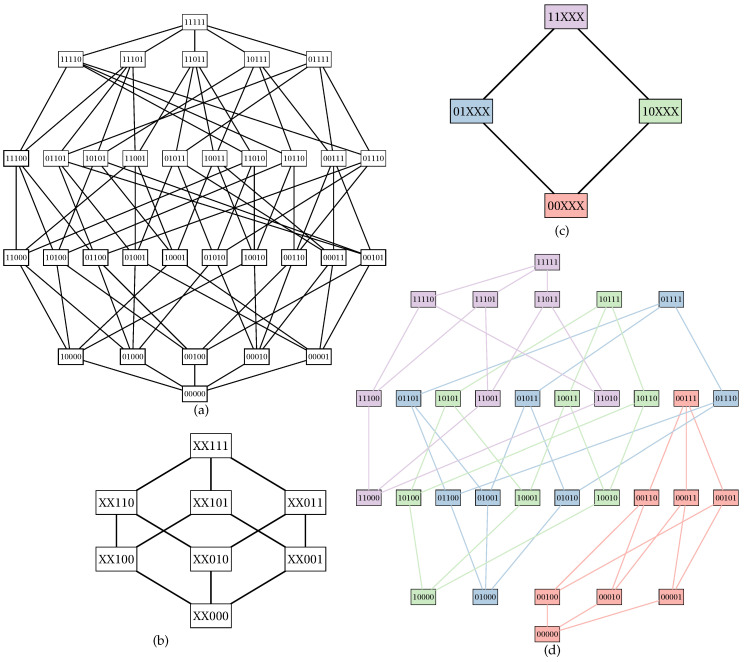
Example of a partition. (**a**) Graph of a Boolean lattice of degree five. (**b**) An outer Boolean lattice defined by fixing the first two elements of the Boolean lattice of degree five; the remaining elements do not care for that partial order and are assigned with ’Xs’. (**c**) One of the four inner Boolean lattices yielded by the partition. (**d**) The partition showed in the Boolean lattice graph in (**a**), where each part is painted with a different color and all edges between parts are excluded.

Let *S* be a non-empty set, and *c* be a decomposable in U-shaped curves cost function. Let us define a subset $S^{\prime }\subseteq S$ named *fixed set*; elements in $S^{\prime }$ are called *fixed points*. The remaining elements in *S* compose a set $\bar{S^{\prime }}=S\backslash S^{\prime }$ named *free set*; elements in $\bar{S^{\prime }}$ are called *free points*. A Boolean lattice (P(S),⊆) can be partitioned into $|S^{\prime }|$ smaller Boolean lattices of degree $|\bar{S^{\prime }}|$ each. For each element $X\in P(S^{\prime })$, we define a small Boolean lattice isomorphic to $\left(P\left(\bar{S^{\prime }}\right),\subseteq \right)$; such lattice associated with *X* is also known as *inner Boolean lattice*. The set $S^{\prime }$ of fixed points can also be used to build a Boolean lattice $\left(P\left(S^{\prime }\right),\subseteq \right)$ called *outer Boolean lattice* (Figure 1).

Let *X* be an element of $P(S^{\prime })$ (i.e., an element of the outer Boolean lattice). We can compute the cost of any element *Y* in the inner Boolean lattice $\left(P\left(\bar{S^{\prime }}\right),\subseteq \right)$ associated with *X* through a wrapper on the original cost function:

(3) $$c_{X}\left(Y\right)=c\left(X\cup Y\right).$$

**Figure 2:**
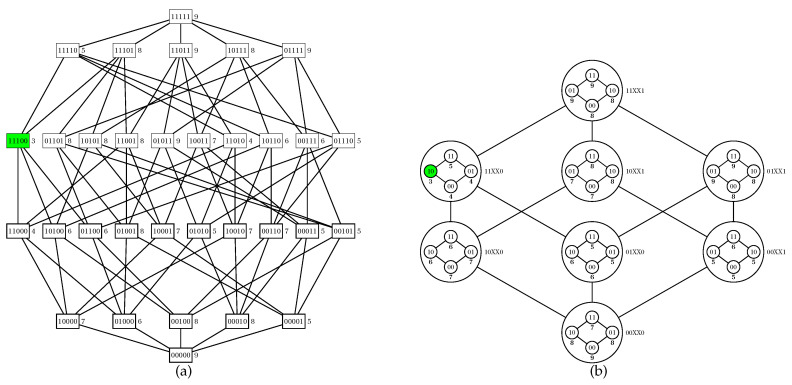
Computing the cost function in a partition. (**a**) An instance of the U-curve problem, where S=11111 and the costs of elements in P(S) are assigned beside the nodes; an element of minimum cost (which is unique in this example) is colored in green. (**b**) A partition scheme where the fixed set is $S^{\prime }:=11001$; each element of the outer Boolean lattice is obtained removing bits from $S^{\prime }$; hence, the third and fourth bits in our string representation do not care for that partial order, thus being assigned with ’Xs’ in this graph and also in the following ones. To compute the cost of element in the inner Boolean lattice associated with a given X, one should set *Y* and then calculate Equation (3): for example, if Y=00100 and X=11000, then $c_{X}\left(Y\right)=c\left(Y\cup X\right)=c\left(00100\cup 11000\right)=c\left(11100\right)=3$.

In Figure 2, we show an example of computation of $c_{X}$ for a partition of a Boolean lattice of degree five.

#### 2.2.2. Dynamics of the PUCS Algorithm

Let *X*, *Y* and *S* be non-empty sets such that X⊆Y⊆S. An *interval*[X,Y] is a collection of elements in P(S) defined as [X,Y]={A∈P(S):X⊆A⊆Y}. The dynamics of the PUCS algorithm consists of walks along the outer Boolean lattice, pruning intervals from the latter whenever it is allowed, that is, when it does not incur risking losing a global minimum. To this end, we make use of two properties of a Boolean lattice-based search space whose cost function is decomposable in U-shaped curves; those properties were already used previously for the designing of optimal algorithms for the U-curve problem [18].

**Proposition** **1.** *Let 〈S,c〉 be an instance of the U-curve problem, and $S^{\prime }\subseteq S$ be a fixed set. Let X and Y be subsets of $S^{\prime }$ such that X⊆Y and X≠Y. If c(X)<c(Y) then it holds that c(X)<c(Z) for any Z∈[Y,S].*

**Proof.**  Suppose that there is a Z∈[Y,S] such that c(X)≥c(Z). Once c(X)<c(Y), it would hold that c(Y)>c(X)≥c(Z), that is, c(Y)>max{c(X),c(Z)}, a contradiction according to the definition of a decomposable in U-shaped curves cost function. □

By the principle of duality, Proposition 1 implies the following result.

**Corollary** **1.** *Let 〈S,c〉 be an instance of the U-curve problem, and $S^{\prime }\subseteq S$ be a fixed set. Let X and Y be subsets of $S^{\prime }$ such that X⊆Y and X≠Y. If c(X)>c(Y) then it holds that c(Y)<c(Z) for any Z∈[∅,X].*

Both Proposition 1 and Corollary 1 establish conditions for pruning of intervals of the Boolean lattice (P(S),⊆) during a walk through the outer Boolean lattice. Therefore, during the execution of PUCS, we test both properties for a given pair X,Y of elements in $\left(P\left(S^{\prime }\right),\subseteq \right)$ such that X⊆Y:

- If c(X)<c(Y), then by Proposition 1 we can remove from the search space elements in [Y,S];
- If $c\left(X\cup \bar{S^{\prime }}\right)>c\left(Y\cup \bar{S^{\prime }}\right)$, then by Corollary 1 we can remove from the search space elements in $\left[\emptyset ,X\cup \bar{S^{\prime }}\right]$.

In Algorithm 1, we present a pseudocode for the PUCS algorithm. In that pseudocode, a walk throughout the outer Boolean lattice is carried out in a **for all** loop between lines 8–25. During an iteration of that loop, we evaluate an element *X* that was recently removed from the current search space U (i.e., a collection of outer Boolean lattice elements that were neither explored nor pruned); *X* is then included into a collection E of explored elements. For each element *Y* from U adjacent to *X* (i.e., an element *Y* from U such that X⊆Y and |Y|=|X|+1 or Y⊆X and |X|=|Y|+1), the two aforementioned conditions are tested, and pruning procedures are carried out accordingly, thus updating U and E; those procedures may result in the replacement of *X* by one of its adjacent elements, therefore changing the evaluated *X* in the **for all** loop and advancing the walk. Eventually, the current search space is fully explored (i.e., U=∅), and we need to look for minima at each inner Boolean lattice associated with elements in E, which is accomplished in line 29. This can be done by either calling the PUCS algorithm itself on the Boolean lattice $\left(P\left(\bar{S^{\prime }}\right),\subseteq \right)$ associated with *X* (i.e., on the instance $\langle \bar{S^{\prime }},c_{X}\rangle$) or using another “base algorithm” (e.g., SFFS or UCS).

**Figure 3:**
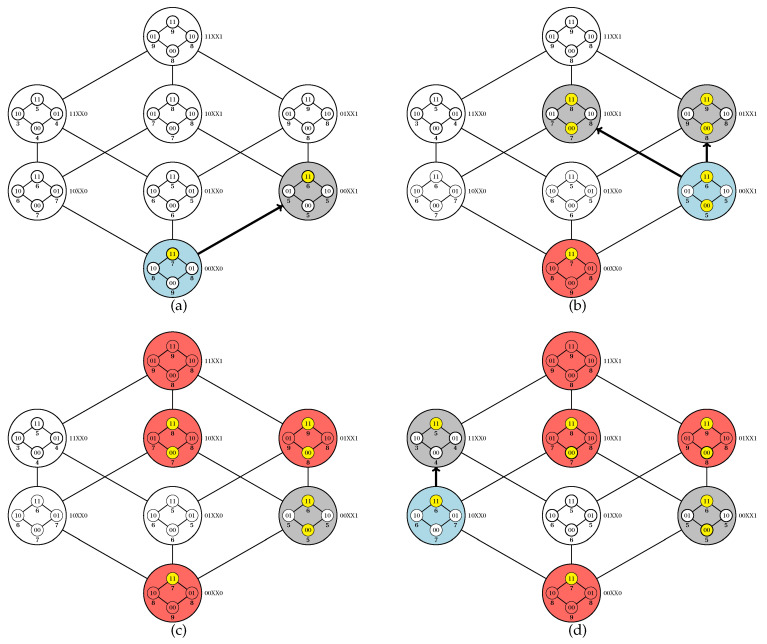
Simulation of the PUCS algorithm (Algorithm 1). Elements of the outer Boolean lattice that are evaluated at a given iteration, explored or pruned from the search space are assigned in blue, gray or red, respectively. Elements of the inner Boolean lattices whose costs were computed are assigned in yellow. (**a**) The search starts at element 00XX0 of the outer Boolean lattice. In the following, element 00XX1 is chosen to be explored; since c(00110)>c(00111), by Corollary 1 the interval [00000,00110] can be removed from the search space. (**b**) Elements 01XX1 and 10XX1 are chosen to be explored; since c(00001)<c(01001) and c(00001)<c(10001), by Proposition 1 the intervals [01001,11111] and [10001,11111] can be removed from the search space. (**c**) There are no possibilities for exploration of the outer Boolean lattice departing from 00XX1. (**d**) Element 10XX0 of the outer Boolean lattice is chosen to initiate a new walk, and explores element 11XX0; since c(10110)>c(11110), then by Corollary 1 the interval [00000,10110] can be removed from the search space. Continue in Figure 4.

**Figure 4:**
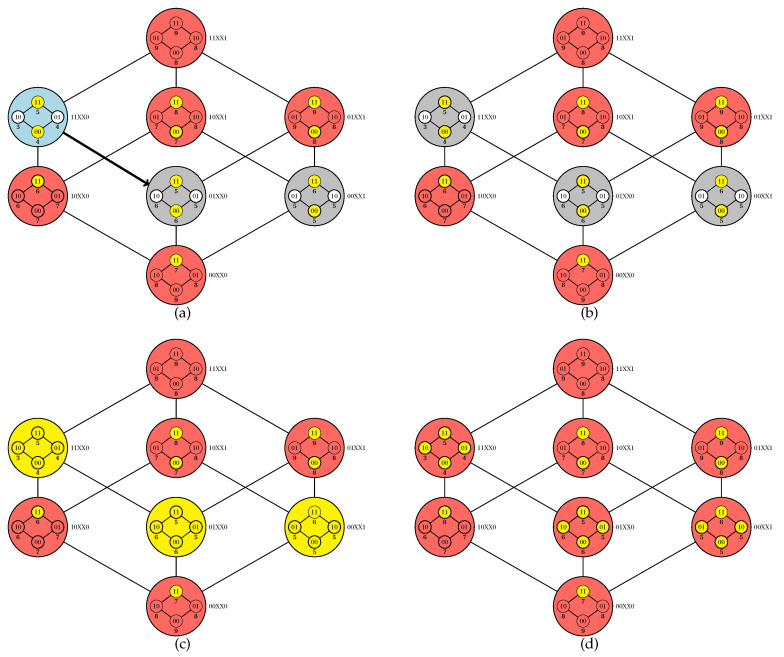
Continuation of the PUCS simulation initiated in Figure 3. (**a**) The element 01XX0 is chosen to be explored from 11XX0; once no pruning is performed, the algorithm chooses 01XX0 for the next iteration (**b**) The element 01XX0 has no unpruned/unexplored adjacent element, thus the iteration ends. (**c**) We solve inner Boolean lattices associated with elements from the outer Boolean lattice that were not pruned from the search space (outer elements in yellow). (**d**) Elements from (P(S),⊆) whose costs were calculated (inner elements in yellow) have 11100 as the one of minimum cost; that also corresponds to the element of minimum cost in the whole Boolean lattice.

Code parallelization is feasible in two procedures: the initiation and execution of a walk (an iteration of the **while** loop between lines 5 and 27) and the exploration of the inner Boolean lattices (line 29). It is noteworthy that the adopted criteria for choosing an element for walk initiation (line 6) or for adjacency exploration (line 8) impact on the algorithm dynamics; for our computational experiments, we adopted a random choice in both cases. In Figure 3 and Figure 4, we provide a simulation of the PUCS algorithm on the instance of the U-curve problem presented in Figure 2a, whose outer Boolean lattice is defined as in Figure 2b.

### 2.3. Experimental Evaluation

We will now present results from computational experiments with the PUCS algorithm. We start this subsection discussing the algorithm parametrization, including its main parameters and how they were set up according to the instance type (Section 2.3.1). In the sequence, we show results with “hard” synthetic data, which were generated taking advantage of the polynomial reduction presented in Equation (1) (Section 2.3.2). We also present results in which we assess the PUCS algorithm robustness when it is dealing with real-world instances, in the context of image filter designing (Section 2.3.3). Finally, we provide results from supervised learning of support-vector machines to classify datasets from the UCI Machine Learning Repository [21] (Section 2.3.4).

To perform the experimental evaluation of the proposed algorithm, we used featsel, a framework coded in C++ especially designed for benchmarking of feature selection algorithms and cost functions [22]. Thus, we implemented PUCS in featsel, using the OpenMP library for code parallelization [23]. Full details about the framework setting up and the adopted parallelization scheme are provided in Materials and Methods (Section 4).
**Algorithm 1** Parallel U-Curve Search (PUCS).**Require:** a non-empty set *S* of features and a decomposable in U-shaped curves cost function *c*    **Ensure:** the elements in P(S) of minimum cost
1:M←∅               ▹M stores elements whose costs were computed2:define a set of fixed points $S^{\prime }\subseteq S$ (hence $\bar{S^{\prime }}$ is the set of free points)3:$U\leftarrow P(S^{\prime })$   ▹U contains unexplored/unpruned elements from the outer Boolean lattice4:E←∅             ▹E contains explored elements from the outer Boolean lattice5:**while**U≠∅**do**6:    remove an element *X* from U7:    E←E∪{X}8:    **for all** Y∈U such that *Y* is adjacent to *X* **do**9:        **if** X⊆Y **and** $c\left(X\cup \bar{S^{\prime }}\right)>c\left(Y\cup \bar{S^{\prime }}\right)$ **then**           ▹ Testing Corollary 110:           remove elements in the interval $\left[\emptyset ,X\cup \bar{S^{\prime }}\right]$ from U and E11:           X←Y12:           E←E∪{X}13:        **else if** X⊆Y **and** c(X)<c(Y) **then**           ▹ Testing Proposition 114:           remove elements in the interval [Y,S] from U and E15:        **else if** Y⊆X**and**c(Y)<c(X)**then**            ▹ Testing Proposition 116:           remove elements in the interval [X,S] from U and E17:           X←Y18:           E←E∪{X}19:        **else if** Y⊆X **and** $c\left(Y\cup \bar{S^{\prime }}\right)>c\left(X\cup \bar{S^{\prime }}\right)$ **then**        ▹ Testing Corollary 120:           remove elements in the interval $\left[\emptyset ,Y\cup \bar{S^{\prime }}\right]$ from U and E21:        **else**22:           X←Y23:           E←E∪{X}24:        **end if**25:    **end for**26:    M←M∪ { elements in P(S) whose costs were computed during this **while** iteration }27:**end while**28:**for all**X∈E**do**29:    M←M∪ { elements of minimum cost in the inner Boolean lattice associated with *X* }30:**end for**31:**return**{M:M∈Mandc(M)isminimum}

#### 2.3.1. Algorithm Parametrization

In our PUCS implementation, we considered three main parameters:

- The maximum recursion depth/level *l* that is allowed when PUCS is recursively called to solve an inner Boolean lattice (line 29 in Algorithm 1). The *l* parameter is an integer ≥1;
- The proportion *p* of features in *S* that will be fixed points. In our implementation, the fixed set size is given by the smallest integer equal or larger than p|S|. The *p* parameter is in the interval (0,1];
- The base algorithm *b* that is called to solve an inner Boolean lattice when the recursion level is equal to *l*. For optimal-search experiments, the *b* parameter was set as U-Curve Branch and Bound (UBB) [17]; in the case of suboptimal-search experiments, *b* was set either as BFS, CHCGA, SFFS or SFS.

The performance of the PUCS algorithm relies heavily on the adopted parametrization; therefore, we will now discuss how to set PUCS parameters according to the instance size. We will consider two types of instances, the small and big ones. Small instances are those that can be solved optimally in a reasonable computational time. Although those instances usually have at most 25 features, naive algorithms such as exhaustive search might already be impractical for solving the problem optimally. On the other hand, big instances are those with more than 25 features, and generally cannot be solved by any optimal algorithm.

##### Small Instances

For small instances, we can set the base algorithm as an optimal solver, more specifically, U-Curve-Branch-and-Bound (UBB), which is a simple, optimal algorithm to solve the U-curve problem [17]. For the other two parameters, *p* and *l*, one should choose them aiming to yield moderate granularity of the partition. A fine-grained partition can create too many parts (up to the point in which every part has only one subset); on the other hand, a coarse-grained partition may not divide the problem enough to allow use of all available computational resources. Therefore, after some assays to assess such trade-off, we decided to set up those parameters as l=1 and p=0.5.

##### Big Instances

**Figure 5:**
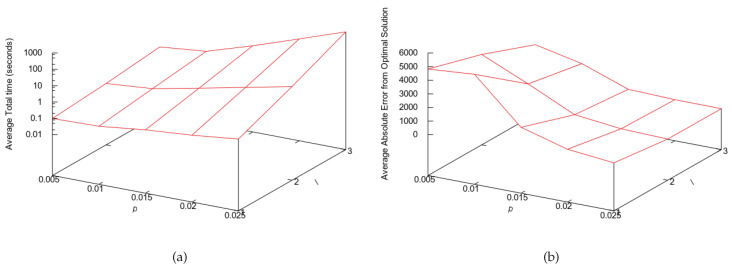
PUCS average performance for synthetic data and different values for parameters *l* and *p*. Each of the 25 considered instances had 200 features and was generated using Equation (1) with random values. The adopted base algorithm was SFS. (**a**) Average required total time per pair 〈l,p〉, in seconds. (**b**) Average absolute error from optimal solution per pair 〈l,p〉.

For big instances, we set the base algorithm with a suboptimal approach, either BFS, CHCGA, SFFS or SFS. When the PUCS base algorithm is optimal, then parameters *l* and *p* can only determine the execution time of the algorithm; however, when the base algorithm is a suboptimal one, *l* and *p* can also interfere on the solution quality, that is, they can determine how close the found solution will be to the global minimum. If the partitioning is fine grained, then more subsets will be visited, producing a solution that tends to be closer to the global minimum. Nevertheless, one cannot increase *l* and *p* arbitrarily, since this would also increase the execution time. In Figure 5, we show the trade-off assessment assay for big instances, where we show how the execution time and solution quality change as we increase the values of *l* and *p*. Based on those results, we decided to set the PUCS parameters for big instances as l=1 and p=10/n, where *n* is number of features of the instance.

#### 2.3.2. Experiments with Synthetic Data

As aforementioned, once Equation (1) is a polynomial reduction from instances of the subset sum problem to instances of the U-curve problem, we used that equation to generate “hard” synthetic instances with *n* features: to this end, one just needs to generate n+1 random integers and use subsets of them to compute Equation (1). We divided our experiments into optimal ones, with instances with up to 18 features, and suboptimal ones, with instances with 30 or more features.

##### Optimal Experiment

In this experiment, we compared the performance of PUCS against UCS, the optimal algorithm for the U-curve problem that was proposed recently [18]. We summarize the yielded results in Table 1. From that table, we remark that even though PUCS needed more calls of the cost function, PUCS outperformed UCS in execution time.

##### Suboptimal Experiment

**Figure 6:**
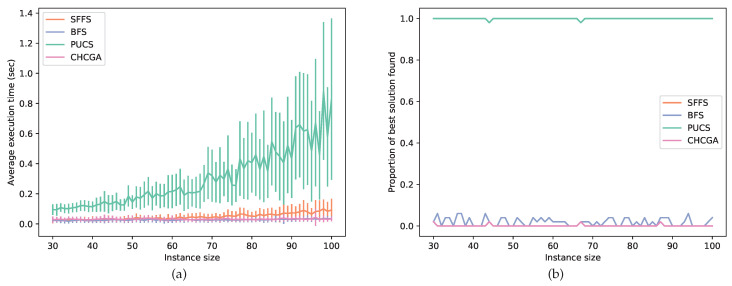
Results from suboptimal experiments with synthetic data. (**a**) Average execution time of the algorithms for different instance sizes (50 instances per size). (**b**) Proportion of times an algorithm found the best solution among the solutions found by all algorithms. For all instance sizes, on average PUCS, BFS, CHCGA and SFFS found the best solution in 99.9 %, 1.9 %, 1.1 % and 1.9 % of the executions, respectively.

In this experiment, the instances had a larger size, ranging from 30 to 100 features; hence, they usually could not be solved by optimal algorithms. For this assay, we set SFS as the PUCS base algorithm and benchmarked this latter with the BFS, CHCGA and SFFS algorithms. Those three latter algorithms were chosen as baselines because each one belongs to a different family of feature selection algorithms, each family with a particular search strategy. In Figure 6, we show a performance comparison between those suboptimal procedures. Observe that PUCS had the worst performance from the computational time point of view. However, for each instance size, PUCS almost always found the best solution among all algorithms.

#### 2.3.3. Assessment of the PUCS Robustness with Real-World Data

During the experiments with synthetic data, the used cost function (Equation (1)) yielded U-shaped curves without violation of the U-curve property (Definition 1). However, when we use real-world data, the chains of the Boolean lattice (P(S),⊆) that represents the search space might have such violations, that is, for a given chain $X_{1}\subseteq X_{2}\subseteq X_{3}$, $X_{i}\in X\subseteq P\left(S\right)$, we might have an element $X_{2}$ such that $c\left(X_{2}\right)>max\left\{c\left(X_{1}\right),c\left(X_{3}\right)\right\}$. In that case, if it also holds that $|X_{3}\left|=\right|X_{2}|+1$ and $|X_{2}\left|=\right|X_{1}|+1$, then we say that such violation is an *oscillation*.

Therefore, to evaluate the PUCS algorithm robustness when facing chain oscillations of real-world instances, we executed feature selection procedures using samples for classifier designing in the context of image processing. A classifier can be used to perform an image filtering (transformation). One important family of image filters is defined by W-operators, morphological operators that are locally defined in windows (i.e., sets of points in a plane) and translation-invariant [24]. To design W-operators, one can firstly select the most relevant features (i.e., a window subset) according to a given criterion; for instance, we can use a penalized mean condition entropy (MCE) cost function that was introduced previously [2], whose formulation is the following: Let *S* be the non-empty set of features, *X* be an element in P(S) and X be a random variable that draws values from P(X), the power set of *X*. Moreover, let *Y* be a discrete random variable representing the class of a given realization x∈P(X) and *P* a probability distribution function. The conditional entropy of *Y* given x is:

(4) $$H\left(Y|X=x\right)=-\sum_{y\in Y}P(Y=y|X=x)\;\log P(Y=y|X=x),$$

Thus, the mean conditional entropy of *Y* given X is:

(5) $$E\left[H\left(Y|X\right)\right]=\sum_{x\in X}H(Y|X=x)\;P(X=x).$$

Once lack of samples might result in underrepresentation of observed pairs 〈y,x〉, we penalize pairs that have a unique observation: This is accomplished by considering them as following a uniform distribution, thus yielding the highest entropy in Equation (4). Thus, the estimation of the penalized mean conditional entropy is:

(6) $$c\left(X\right)=\hat{E}\left[H\left(Y|X\right)\right]=\frac{N}{t}+\sum_{x\in X:\hat{P}\left(x\right)>\frac{1}{t}}\hat{H}\left(Y|X=x\right)\;\hat{P}\left(X=x\right),$$

where *N* is the frequency that X has a single sample occurrence and *t* is the number of samples.

To accomplish conditional entropy-based feature selection in the W-operator filter designing, we used the same training set of a previous study [18]. In that study, it was produced ten pairs 〈observed,ideal〉 of binary images. Each observed image was produced by covering its respective ideal image with 30 % of uniformly distributed, salt-and-pepper noise. For each pair of binary images and for each window size ranging from 7 to 17, a window was screened throughout the image pair, thus producing the samples for the feature selection procedures. Those produced instances can be retrieved from an online repository [25].

The obtained results are summarized in Table 2 and Table 3. In despite of the number of oscillations increased with the increase of the instance size, the PUCS algorithm almost always could find an optimal solution (Table 2). There was an exception for a single instance of size 17, for which PUCS could not find the global optimum; however, when we replaced UBB for UCS as the base algorithm, PUCS could also find the global minimum for all ten instances of size 17. On the other hand, the suboptimal algorithms (BFS, CHCGA and SFFS) had a decrease in finding the global minimum as a function of instance size, a phenomenon that was already expected in that type of assay.

From the computational point of view, PUCS required much less computational time than UCS; for example, for instances of size 17, PUCS required on average only 3.19% and 1.45% of computational required by exhaustive search (ES) and UCS, respectively (Table 3). On the other hand, although the number of cost function calls made by PUCS was only 42.09% of the one required by ES, it was 40.63% higher than the one required by UCS. However, when we replaced UBB for UCS as the base algorithm, the PUCS average number of calls of cost function for instances of size 17 was 44913.3 ± 5015.9, which is only 13.57 % higher than the one of UCS.

#### 2.3.4. Experiments with Machine Learning Datasets

Whereas in the previous section we evaluated the robustness of the PUCS algorithm in mean conditional entropy-based feature selection searches, now we assess the performance of PUCS and other algorithms (BFS, CHCGA, SFFS and UCS) in the feature selection step for the designing of Support-Vector Machines (SVMs). SVMs are classifiers that create a decision boundary in the feature space to separate data into two different classes; the boundaries are chosen in a way that they tend to have a maximum distance to the nearest points of both classes [26].

The experiment started with feature selection procedures on discrete Machine Learning datasets, using the penalized mean conditional entropy (Equation (6)) as the cost function. After feature selection, we then used the LIBSVM open-source library to train SVM models and perform cross-validation [27]. For instances where there are more than two classes, the library creates a binary classifier for each pair of classes and use a consensus scheme to classify new data. We used linear SVMs with regularization parameter (i.e., a parameter that changes boundary margin sizes) set as 100. We used 10-fold cross validation (CV) for datasets with less than 100 samples, and leave-one-out for other datasets.

The used datasets were downloaded from the University of California Irvine (UCI) Machine Learning Repository [21]. A total of five datasets were retrieved, with different number of features, classes and samples (Table 4). All datasets were converted to the featsel framework format and some of them had their features discretized for feature selection and classification. In Table 5, we show, for each algorithm and for each dataset, the number of selected features and also the cross-validation (CV) error of the trained SVM classifier. In that experiment, the SVM trained using features selected with PUCS yielded a smallest CV error in the two largest datasets (“Lung cancer” and “Hill Valley”). Moreover, a PUCS feature selection procedure with a given base algorithm yielded a classifier with a CV error equal or smaller to the one of the same base algorithm executed alone in 10 out of 15 procedures (66.67 %). Similarly, the former procedure selected a feature subset of size equal or smaller to the one of the latter also in 10 out of 15 procedures.

For the smaller datasets, namely Breast Cancer, Wine and Zoo, we verified the number of oscillations present in their respective search spaces, which are 94, 1,832 and 226,941, respectively. Hence, we also performed feature selection on those datasets with an exhaustive search (ES). Those procedures yielded SVM classifiers with CV errors for Breast Cancer, Wine and Zoo of 4.1 %, 2.8 % and 6.9 %, respectively. The number of selected features were, respectively, five, four and six. Importantly, when we applied PUCS on those same three datasets with an optimal base algorithm (UBB), the selected subsets were the same of ES, thus yielding the same CV errors and number of selected features of this latter optimal procedure.

## 3. Discussion

Solving a feature selection problem through an approximation to the U-curve problem is an effective strategy that was demonstrated previously in a wide range of applications such as dataset classification and designing of morphological operators [16,17,18]. However, in all those instances, the computational performance of the U-curve-based algorithm was exponential on the number of features, thus hindering the scalability of the algorithms proposed in those works. Here, we demonstrated that such issue was not due to poorly designed algorithms, since we proved that in fact the U-curve problem is NP-hard (Theorem 1).

Once solving an instance of the U-curve problem probably is not feasible in polynomial time, we presented in this work the Parallel U-Curve Search (PUCS), a new algorithm for that problem that was designed with properties that mitigate the aforementioned scalability issues. PUCS allows us to take advantage of the highly symmetrical structure of the Boolean lattice representation of the search space to produce a partition of the latter (Figure 1); each part, known as “inner Boolean lattice”, can be solved independently in a parallelized fashion. Moreover, the organization of those parts in a partial order results in a lattice that we called “outer Boolean lattice”: such structure can be explored through walks, in which pruning conditions can be evaluated; if the removal of a search space interval is applicable (according to Proposition 1 or Corollary 1), then one or more parts can be discarded unsolved, thus enhancing the computational performance of the algorithm.

To experimentally validate the new algorithm, we coded PUCS in featsel, a C++ framework for benchmarking of feature selection algorithms and cost functions [22]. We carried out computational assays with synthetic data, where we evaluated the computational time requirement of PUCS in comparison with UCS, which is the latest proposed algorithm for the U-curve problem [18]. The optimal experiment showed us that PUCS required just a small fraction of the time spent by UCS: For example, for instances of size 18, on average PUCS required less than 0.1% of the UCS time (Table 1). However, PUCS computed more times the cost function than UCS: for instances of size 18, PUCS calculated it around four times more often; this could be due to the choice of base algorithm (UBB), which is is known to compute more often the cost function than UCS [32]. In the suboptimal experiment, we compared PUCS against UCS and other three golden standard algorithms, namely BFS, CHCGA and SFFS. In that assay, the solution found by other algorithms were usually worse than the solution found by PUCS (Figure 6); the superior PUCS performance might come from the partition scheme, which provides a better coverage of different regions of the search space.

Once in experiments with synthetic data all instances do not violate the U-curve property (Definition 1), we evaluated the PUCS algorithm robustness and optimality when it is dealing with real-world data, since those latter might have violations of that property. Indeed, during the feature selection step of W-operator image filter designing, the number of oscillations per element in the search space increases as a function of instance size (Table 2): for example, for instances of size 7 (i.e., search space with $2^{7}$ elements) the average number of oscillations per element is 0.03, whereas for instances of size 17 (i.e., search space with $2^{17}$ elements) that number is 1.03. Nevertheless, the tested optimal algorithms for the U-curve problem have a robustness in respect to those oscillations, returning the global minimum for almost all evaluated instances: the only exception was one instance with 17 features, for which PUCS using UBB as base algorithm could not find the global minimum; once that situation was circumvented when we used UCS instead of UBB as the base algorithm for PUCS, UCS probably is a more robust optimal base algorithm for PUCS than UBB. In those assays in the context of W-operator designing, we also confirmed that the code parallelization of PUCS provides, from the computational point of view, a more efficient feature selection procedure than both exhaustive search and UCS (Table 3).

We also executed computational assays with Machine Learning datasets, where we evaluated the PUCS algorithm in the feature selection step of Support-Vector Machine (SVM) designing for classification of a number of datasets (Table 4). In those experiments, we employed the PUCS algorithm with three different parametrizations, each one using one of the benchmarked feature selection algorithms (BFS, CHCGA and SFFS). The idea of that experiment was to show whether the usage of PUCS, combined with one of those renowned suboptimal algorithms, could improve their performance in the feature selection step of classifier designing. Indeed, in two-thirds of those experiments, PUCS combined with one of the mentioned suboptimal algorithm yielded a CV error that was equal to or smaller than the one yielded by the standalone run of the respective suboptimal algorithm (Table 5); the same proportion was verified when we analyzed the selected number of features, which is related to a reduction of the model complexity (Table 5). The improvement provided by PUCS might come from the aforementioned property, whose usage allows a more structured exploration of the search space. Besides, the walk through the outer Boolean lattice that represents the search space also allows pruning proceedings, thus preventing the base algorithm from exploring unnecessary regions of the search space and also from computing the cost of a same element twice or more times. Finally, for the smaller datasets (Breast Cancer, Wine and Zoo), we verified that, despite the presence of oscillations in their respective search spaces, the PUCS algorithm, when using an optimal solver for the U-curve problem (UBB) as the base algorithm, could always find the subset that minimizes the penalized mean conditional entropy. The slightly higher CV errors yielded by those optimum subsets in comparison to the smallest CV errors depicted in Table 5 could be attributed to the constraints on the hypothesis space of SVM models, which is regulated by the learning algorithm parametrization; for example, changes in the regularization parameter and/or in the used kernel (e.g., from linear to polynomial) induce significant changes on the model validation results.

The effectiveness of the proposed algorithm to tackle feature selection in instances with greater levels of interactions among features (e.g., the prediction of intrinsically multivariate genes) is a subject that we intend to investigate in this research line. Another possibility would be the application of PUCS on very large datasets; for instance, on the CASIA Chinese handwriting database, which contains about 3.9 million samples and 7,356 classes [33]. In particular, it would be interesting to compare the performance of PUCS against minimum redundancy, maximum relevance (MRMR) methods that are often used to solve large problems [4], which include approaches such as the extended MRMR and the SpecCMI algorithm [34,35].

## 4. Materials and Methods

### 4.1. Algorithm Implementation

The PUCS algorithm was coded in C++, using featsel, a framework for benchmarking of feature selection algorithms and cost functions [22]. To carry the computational experiments whose results were depicted in Section 2.3, we also implemented in featsel the subset sum cost function (Equation (1)). Implementations for the penalized MCE cost function (Equation (6)), as well as for the BFS, CHCGA, ES, SFFS, SFS, UBB and UCS algorithms were already available at the featsel repository, since they were used in previous studies [17,18]; the parametrization of those algorithms was also the same employed previously: SFFS with parameter delta equal to 3; CHCGA with a population size equal to 50, difference threshold equal to $\frac{\left|S\right|}{4}$ and divergence rate equal to 0.35; BFS with accuracy equal to $10^{-5}$ and number of expansions equal to 5.

In the remainder of this subsection, we will provide details about key points in the implementation of the PUCS algorithm.

#### 4.1.1. Search Space Management

When an element from the outer Boolean lattice is either explored or pruned it is necessary to remove it from the collection U (Algorithm 1). Once initially the size of U is equal to the power set of the set of fixed points, that is, $|U|=|P\left(S^{\prime }\right)|=2^{|S^{\prime }|}$, the data structure to be used must not represent explicitly (at least not initially) each element in U. Besides, the chosen data structure must also be efficient for removals and queries. Therefore, we chose to use Reduced Ordered Binary Decision Diagrams (ROBDDs), a data structure that is useful for compressed representation of sets [36].

#### 4.1.2. Code Parallelization

To parallelize the PUCS code, we instructed the compiler that the partitioning and walk on the outer Boolean lattice should be made by a master thread, while the solving of each inner Boolean lattice should be computed by other worker threads. Such instruction was done using the OpenMP API [23]; OpenMP allows us to annotate the code with directives that indicate to the compiler how blocks of code can be processed in parallel. Therefore, it is possible to adjust the work distribution among tasks (i.e., walking on the outer Boolean lattice or solving the inner Boolean lattices) and threads, with minimal need of communication and synchronization between workers.

### 4.2. Computational Resources

Computational experiments in which computational time was evaluated were conduced either in a server with an Intel^®^ Xeon^®^ CPU E5-2690 processor, with 32 processing cores, 256 GB of RAM and Ubuntu operating system (Figure 5 and Figure 6 and Table 1) or in a server with an AMD Opteron^™^ processor, 64 processing cores, 256 GB RAM and Ubuntu operating system (Table 2 and Table 3).

### 4.3. Source Code Availability

Source code of the PUCS algorithm is under GNU GPLv3 license and can be obtained for free at the featsel GitHub repository: github.com/msreis/featsel.

## Figures and Tables

**Table 1:** Results from optimal experiments with synthetic data. For each instance size and algorithm, it is given the mean and standard deviation of 50 independent executions.

Instance Size		Required Computational Time (sec)		# Cost Function Calls	
|S|	$2^{\left|S\right|}$	UCS	PUCS	UCS	PUCS
10	1024	0.02 ± 0.01	0.02 ± 0.01	311.1 ± 134.9	794.6 ± 271.5
11	2048	0.04 ± 0.03	0.05 ± 0.02	463.6 ± 322.7	1352.5 ± 907.5
12	4096	0.17 ± 0.17	0.08 ± 0.03	941.0 ± 604.2	2801.9 ± 1607.6
13	8192	0.45 ± 0.64	0.08 ± 0.03	1232.6 ± 951.9	4199.2 ± 2736.7
14	16384	2.73 ± 3.26	0.12 ± 0.08	2617.0 ± 2448.2	7625.6 ± 6534.1
15	32768	6.62 ± 12.21	0.10 ± 0.07	3048.4 ± 3738.7	11108.1 ± 10973.1
16	65536	19.94 ± 38.92	0.20 ± 0.12	5794.6 ± 6060.9	20530.8 ± 19061.4
17	131072	137.00 ± 197.40	0.27 ± 0.15	12166.9 ± 11982.3	51161.2 ± 42987.5
18	262144	431.21 ± 764.93	0.38 ± 0.29	19459.6 ± 20107.7	82169.4 ± 78727.2

**Table 2:** Results from experiments with real-world data from mean conditional entropy-based feature selection step of image filter designing. For each instance size and algorithm, it is given the number of times a given algorithm reaches the global optimum of a given instance. For each instance size (ten instances per size), it is also given the average and standard deviation for the number of oscillations, that is, the number of violations of the U-curve property. ES stands for exhaustive search.

Instance		# Oscillations	# Optimal Solution					
|S|	$2^{\left|S\right|}$		PUCS	SFFS	CHCGA	BFS	UCS	ES
7	128	4.0 ± 3.1	10	4	5	6	10	10
8	256	21.7 ± 21.4	10	5	7	3	10	10
9	512	93.1 ± 83.9	10	6	9	3	10	10
10	1024	212.4 ± 207.7	10	4	5	3	10	10
11	2048	325.1 ± 343.1	10	5	4	3	10	10
12	4096	1765.6 ± 1357.4	10	3	2	3	10	10
13	8192	3743.7 ± 3497.2	10	4	3	3	10	10
14	16384	9137.6 ± 12037.6	10	3	5	2	10	10
15	32768	16839.2 ± 24126.7	10	4	2	2	10	10
16	65536	39539.2 ± 64501.1	10	4	0	0	10	10
17	131072	135396.2 ± 175009.2	9	3	0	1	10	10

**Table 3:** Results from experiments with real-world data from mean conditional entropy-based feature selection step of image filter designing. For each instance size and algorithm, it is given the mean and standard deviation of executions on 10 different instances. ES stands for exhaustive search.

**Instance**		**Required Computational Time (sec)**					
|S|	$2^{\left|S\right|}$	**PUCS**	**SFFS**	**CHCGA**	**BFS**	**UCS**	**ES**
7	128	0.07 ± 0.01	0.10 ± 0.02	0.14 ± 0.04	0.03 ± 0.01	0.07 ± 0.02	0.13 ± 0.03
8	256	0.14 ± 0.04	0.22 ± 0.08	0.37 ± 0.08	0.05 ± 0.01	0.19 ± 0.04	0.31 ± 0.07
9	512	0.23 ± 0.06	0.38 ± 0.14	0.64 ± 0.18	0.07 ± 0.01	0.48 ± 0.11	0.78 ± 0.18
10	1024	0.49 ± 0.11	0.60 ± 0.25	0.80 ± 0.21	0.09 ± 0.02	1.11 ± 0.25	1.86 ± 0.43
11	2048	0.72 ± 0.15	0.76 ± 0.26	1.01 ± 0.22	0.13 ± 0.03	2.91 ± 0.76	4.38 ± 0.99
12	4096	1.36 ± 0.22	0.85 ± 0.42	0.92 ± 0.29	0.16 ± 0.03	7.16 ± 1.41	10.17 ± 2.30
13	8192	2.50 ± 0.99	1.12 ± 0.50	1.08 ± 0.30	0.20 ± 0.04	17.99 ± 2.93	23.69 ± 5.22
14	16384	4.73 ± 1.08	1.59 ± 0.99	1.31 ± 0.44	0.25 ± 0.05	50.66 ± 8.69	55.52 ± 12.10
15	32768	7.66 ± 1.93	1.64 ± 0.55	1.45 ± 0.32	0.31 ± 0.06	156.94 ± 34.58	125.07 ± 27.77
16	65536	13.49 ± 2.17	1.98 ± 1.00	1.45 ± 0.43	0.37 ± 0.08	526.16 ± 172.39	286.58 ± 61.79
17	131072	20.29 ± 5.61	2.21 ± 0.59	1.65 ± 0.36	0.41 ± 0.09	1396.78 ± 428.62	636.45 ± 142.96
**Instance**		# **Cost Function Calls**					
|S|	$2^{\left|S\right|}$	**PUCS**	**SFFS**	**CHCGA**	**BFS**	**UCS**	**ES**
7	128	107.4 ± 18.7	127.1 ± 2.8	128.0 ± 0.0	29.0 ± 0.0	52.8 ± 7.7	128.0
8	256	231.7 ± 43.8	222.3 ± 45.8	252.5 ± 7.7	37.0 ± 0.0	123.7 ± 22.8	256.0
9	512	442.9 ± 91.2	323.3 ± 122.1	368.5 ± 58.4	45.9 ± 0.3	256.5 ± 21.3	512.0
10	1024	1003.0 ± 78.3	399.6 ± 114.1	387.8 ± 63.9	55.8 ± 0.4	499.8 ± 45.1	1024.0
11	2048	1939.0 ± 85.7	479.0 ± 120.8	436.1 ± 36.2	65.7 ± 0.9	1067.8 ± 89.4	2048.0
12	4096	3499.1 ± 326.5	461.4 ± 101.0	353.3 ± 68.6	75.4 ± 1.3	2060.7 ± 90.7	4096.0
13	8192	6042.5 ± 1045.2	563.1 ± 205.1	362.1 ± 59.0	85.6 ± 1.3	3985.1 ± 129.6	8192.0
14	16384	11176.7 ± 1710.8	704.8 ± 362.3	395.7 ± 38.9	96.2 ± 3.3	7312.0 ± 527.5	16384.0
15	32768	20727.9 ± 2515.0	709.3 ± 234.2	408.8 ± 33.3	107.5 ± 2.4	13444.1 ± 1429.5	32768.0
16	65536	36144.5 ± 5161.6	803.9 ± 404.4	364.7 ± 38.2	117.7 ± 4.3	23940.4 ± 3753.1	65536.0
17	131072	55168.0 ± 8616.3	835.6 ± 227.6	381.9 ± 33.9	126.7 ± 2.2	39228.4 ± 3713.5	131072.0

**Table 4:** Main statistics of all datasets used in the SVM designing experiments.

Dataset	# Features	# Classes	# Samples	Reference Paper
Breast cancer	10	2	699	[28]
Wine	13	3	178	[29]
Zoo	15	7	101	[21]
Lung Cancer	56	3	32	[30]
Hill Valley	100	2	606	[31]

**Table 5:** Results of SVM classifiers designed with features selected by different algorithms. For each dataset and algorithm, it is given the mean and standard deviation of: cross-validation (CV) error; number of selected features. Those statistics are results from 10 independent executions. In the case of PUCS, the name between parenthesis indicates the used base algorithm. “All” means that all features were considered for SVM designing. An underlined value indicates that, for a given dataset, an algorithm had, among all algorithms, the lowest CV or the smallest subset of selected features. A bold value indicates that, for a given dataset, PUCS using a base algorithm B had a better performance than the average standalone executions of B, where B is either BFS, CHCGA or SFFS.

	**Cross-Validation Error (%)**						
**Dataset**	**SFFS**	**PUCS**	**CHCGA**	**PUCS**	**BFS**	**PUCS**	**All**
		**(SFFS)**		**(CHCGA)**		**(BFS)**	
B. cancer	4.1 ± 0.0	4.3 ± 0.2	4.0 ± 0.2	4.1 ± 0.1	4.0 ± 0.0	4.1 ± 0.1	4.0
Wine	2.8 ± 0.0	2.8 ± 0.0	6.3 ± 3.4	**5.8 ± 3.2**	2.8 ± 0.0	2.8 ± 0.0	1.1
Zoo	6.9 ± 0.0	6.9 ± 0.0	6.9 ± 2.7	**5.8 ± 1.9**	5.0 ± 0.0	5.4 ± 0.7	8.9
Lung cancer	37.5 ± 0.0	**36.3 ± 0.0**	63.1 ± 6.2	**57.2 ± 6.1**	37.5 ± 0.0	38.1 ± 2.0	58.4
Hill Valley	44.1 ± 0.0	**44.0 ± 0.3**	42.5 ± 0.5	**42.3 ± 0.5**	43.6 ± 0.0	43.6 ± 0.1	42.7
	# **Selected Features**						
**Dataset**	**SFFS**	**PUCS**	**CHCGA**	**PUCS**	**BFS**	**PUCS**	**All**
		**(SFFS)**		**(CHCGA)**		**(BFS)**	
B. cancer	5.0 ± 0.0	**4.7 ± 0.5**	5.4 ± 0.5	5.4 ± 0.5	4.0 ± 0.0	4.2 ± 0.4	10
Wine	4.0 ± 0.0	4.0 ± 0.0	3.8 ± 0.4	4.0 ± 0.0	4.0 ± 0.0	4.0 ± 0.0	13
Zoo	6.0 ± 0.0	6.0 ± 0.0	6.5 ± 0.5	6.8 ± 0.8	7.0 ± 0.0	**6.9 ± 0.3**	15
Lung cancer	4.0 ± 0.0	4.2 ± 0.6	21.6 ± 5.4	**5.5 ± 7.4**	4.0 ± 0.0	4.0 ± 0.0	56
Hill Valley	4.0 ± 0.0	4.1 ± 0.3	42.7 ± 3.9	**41.5 ± 5.6**	5.0 ± 0.0	**4.9 ± 0.3**	100

## Abbreviations

The following abbreviations are used in this manuscript:

BFS	Best-First Search
CASIA	Chinese Academy of Sciences, Institute of Automation
CFS	Correlation-based Feature Selection
CMI	Conditional Mutual Information
CHCGA	Cross generational elitist selection, Heterogeneous recombination, and Cataclysmic mutation GA
DFS	Depth-First Search
ES	Exhaustive Search
EMRMR	Extended MRMR
GA	Genetic Algorithm
iUBB	improved U-curve Branch and Bound
MCE	Mean Conditional Entropy
MRMR	Minimum redundancy, maximum relevancy
PUCS	Parallel U-Curve Search
ROBDD	Reduced Ordered Binary Decision Diagram
SFFS	Sequential Forward Floating Selection
SFS	Sequential Forward Selection
SpecCMI	Spectral relaxation for solving CMI-based quadratic programming feature selection
SVM	Support-Vector Machine
UBB	U-curve Branch and Bound
UCS	U-Curve Search

## References

[1] Dyson F. (2004). A meeting with Enrico Fermi. Nature.

[2] Martins D., Cesar R., Barrera J. (2006). W-operator window design by minimization of mean conditional entropy. Pattern Anal. Appl..

[3] Hall M.A. (2000). Correlation-Based Feature Selection of Discrete and Numeric Class Machine Learning.

[4] Hanchuan P., Fuhui L., Ding C. (2005). Feature selection based on mutual information criteria of max-dependency, max-relevance, and min-redundancy. IEEE Trans. Pattern Anal. Mach. Intell..

[5] Kalai A.T., Mansour Y., Verbin E. On Agnostic Boosting and Parity Learning. Proceedings of the Fortieth Annual ACM Symposium on Theory of Computing.

[6] Martins D.C., Braga-Neto U.M., Hashimoto R.F., Bittner M.L., Dougherty E.R. (2008). Intrinsically Multivariate Predictive Genes. IEEE J. Sel. Topics Signal Process..

[7] Chen Y., Miao D., Wang R. (2010). A rough set approach to feature selection based on ant colony optimization. Pattern Recognit. Lett..

[8] Jović A., Brkić K., Bogunović N. A review of feature selection methods with applications. Proceedings of the 2015 38th International Convention on Information and Communication Technology, Electronics and Microelectronics (MIPRO).

[9] Chandrashekar G., Sahin F. (2014). A survey on feature selection methods. Comput. Electr. Eng..

[10] Marill T., Green D. (1963). On the effectiveness of receptors in recognition systems. IEEE Trans. Inf. Theory.

[11] Pudil P., Novovicová J., Kittler J. (1994). Floating search methods in feature selection. Pattern Recognit. Lett..

[12] Whitney A.W. (1971). A Direct Method of Nonparametric Measurement Selection. IEEE Trans. Comp..

[13] Xue B., Zhang M., Browne W.N., Yao X. (2016). A Survey on Evolutionary Computation Approaches to Feature Selection. IEEE Trans. Evol. Comput..

[14] Eshelman L.J. (1991). The CHC adaptive search algorithm: How to have safe search when engaging in nontraditional genetic recombination. Foundations of Genetic Algorithms.

[15] Frank E., Hall M., Trigg L., Holmes G., Witten I.H. (2004). Data mining in bioinformatics using Weka. Bioinformatics.

[16] Ris M., Barrera J., Martins D. (2010). U-curve: A branch-and-bound optimization algorithm for U-shaped cost functions on Boolean lattices applied to the feature selection problem. Pattern Recognit..

[17] Atashpaz-Gargari E., Reis M., Braga-Neto U., Barrera J., Dougherty E. (2018). A fast Branch-and-Bound algorithm for U-curve feature selection. Pattern Recognit..

[18] Reis M.S., Estrela G., Ferreira C.E., Barrera J. (2019). Optimal Boolean lattice-based algorithms for the U-curve optimization problem. Inf. Sci..

[19] Banon G.J.F., Barrera J. (1991). Minimal Representations for Translation-Invariant Set Mappings by Mathematical Morphology. SIAM J. Appl. Math..

[20] Cormen T., Leiserson C., Rivest R., Stein C. (2001). Introduction to Algorithms.

[21] Dua D., Graff C. (2017). UCI Machine Learning Repository. mlr.cs.umass.edu/ml.

[22] Reis M.S., Estrela G., Ferreira C.E., Barrera J. (2017). featsel: A framework for benchmarking of feature selection algorithms and cost functions. SoftwareX.

[23] Dagum L., Menon R. (1998). OpenMP: An industry-standard API for shared-memory programming. Comput. Sci. Eng..

[24] Barrera J., Terada R., Hirata R., Hirata N. (2000). Automatic programming of morphological machines by PAC learning. Fund. Inform..

[25] Reis M.S. (2017). W-Operator Filter: A Set of Programs to Design and Apply W-Operator Filters on Noisy Binary Images. github.com/msreis/W-operator-filter.

[26] Cortes C., Vapnik V. (1995). Support-vector networks. Mach. Learn..

[27] Chang C.C., Lin C.J. (2011). LIBSVM: A Library for Support Vector Machines. ACM Trans. Intell. Syst. Technol..

[28] Wolberg W.H., Mangasarian O.L. (1990). Multisurface method of pattern separation for medical diagnosis applied to breast cytology. Proc. Natl. Acad. Sci. USA.

[29] Aeberhard S., Coomans D., De Vel O. (1994). Comparative analysis of statistical pattern recognition methods in high dimensional settings. Pattern Recognit..

[30] Hsieh T.H., Chen J.J.J., Chen L.H., Chiang P.T., Lee H.Y. (2011). Time-course gait analysis of hemiparkinsonian rats following 6-hydroxydopamine lesion. Behav. Brain Res..

[31] Hong Z.Q., Yang J.Y. (1991). Optimal discriminant plane for a small number of samples and design method of classifier on the plane. Pattern Recognit..

[32] Reis M.S. (2012). Minimization of Decomposable in U-shaped Curves Functions Defined on Poset Chains–Algorithms and Applications. Ph.D. Thesis.

[33] Liu C., Yin F., Wang D., Wang Q. CASIA Online and Offline Chinese Handwriting Databases. Proceedings of the 2011 International Conference on Document Analysis and Recognition.

[34] Herman G., Zhang B., Wang Y., Ye G., Chen F. (2013). Mutual information-based method for selecting informative feature sets. Pattern Recognit..

[35] Nguyen X.V., Chan J., Romano S., Bailey J. Effective Global Approaches for Mutual Information Based Feature Selection. Proceedings of the 20th ACM SIGKDD International Conference on Knowledge Discovery and Data Mining.

[36] Bryant R.E. (1986). Graph-Based Algorithms for Boolean Function Manipulation. IEEE Trans. Comput..

